# The Effect of Sethoxydim Herbicide on the Physiological Parameters, Photosynthetic Enzymes and Antioxidant System in Foxtail Millet

**DOI:** 10.3390/plants15030511

**Published:** 2026-02-06

**Authors:** Lizhi Li, Tao Jing, Xikai Lin, Yue Zhuang, Yiru Wang, Dan Liu, Huiling Du, Xiaorui Li

**Affiliations:** 1 College of Agriculture, Shanxi Agricultural University/Key Laboratory of Sustainable Dryland Agriculture of Shanxi Province, Taiyuan 030031, China; lilitchi@outlook.com (L.L.); 19834549920@163.com (T.J.); nightboxbox@163.com (X.L.); zyfc0112@163.com (Y.Z.); 2 College of Agriculture, Shanxi Agricultural University, Special Orphan Crops Research Center of the Loess Plateau, MARA, Taigu 030800, China; wyrwyr2000@163.com (Y.W.); liudan517811721@163.com (D.L.)

**Keywords:** foxtail millet, sethoxydim, agronomic traits, photosynthetic characteristics, antioxidant enzymes

## Abstract

Foxtail millet (*Setaria italica* L.) possesses characteristics such as strong stress tolerance and high yield. However, weeds compete with foxtail millet, leading to reduced crop yield, degraded quality, and even the promotion of pest and disease spread. Chemical weed control is currently the most practical and feasible method for preventing weed damage in foxtail millet production, but herbicides can harm the main crop, resulting in reduced yield. To investigate the effects of sethoxydim on the growth and development of foxtail millet, this experiment adopted a pot design, setting four concentration gradients for foliar spraying: 0.75, 1.5 (recommended dosage), 3 and 6 L of active ingredient per hectare (L ai ha^−1^). Sethoxydim treatment hindered electron transport in photosynthesis, leading to a decrease in adenosine triphosphate synthesis and consequently a decline in the photosynthetic parameters of both photosystem I and photosystem II. Meanwhile, the activities and related gene expression of phosphoenolpyruvate carboxylase (PEPC), NADP-malate dehydrogenase (NADP-MDH) and pyruvate phosphate dikinase (PPDK) all showed a decreasing trend. In contrast, the activities and related gene expression of NADP-malic enzyme (NADP-ME) and ribulose-1,5-bisphosphate carboxylase/oxygenase (Rubisco); the contents of soluble protein and soluble sugar; and the activities of antioxidant enzymes including malondialdehyde (MDA), superoxide dismutase (SOD), peroxidase (POD), and catalase (CAT), along with their related gene expression, exhibited a trend of first increasing and then decreasing, reaching their peak at a dosage of 1.5 L ai ha^−1^ (T2 treatment group). Meanwhile, the continuous rise in O_2_^·^−^^ and H_2_O_2_ contents indicated enhanced accumulation of reactive oxygen species (ROS) in plants under herbicide stress. These results show that at the recommended dosage, although sethoxydim causes certain damage to foxtail millet, the plant can maintain certain photosynthetic functions and physiological stability through self-regulation and gradually return to normal.

## 1. Introduction

Foxtail millet (*Setaria italic* L.) originated in Northern China [1]. It has high tolerance to abiotic stresses, especially drought, salt and poor soil, and it is a promising model plant for C4 grasses [2,3]. In addition, it has many nutritional and antioxidant functions. These advantages make foxtail millet an ideal crop to solve food challenges in semi-arid regions in Asia [4].

The key factor impacting its production is weed infestation. Weeds compete for nutrients, water and light, thereby adversely influencing the growth, yield and quality of crops. In recent years, herbicides have been extensively utilized in agriculture to effectively control weeds. Herbicides are effective for removing relevant specific weeds [5]. Nevertheless, herbicides can also affect the growth and development of plants, which is mainly related to the chemical and oxidative damage caused by herbicides.

Sethoxydim is an acetyl-CoA carboxylase (ACCase) inhibitor and causes chloroplast bleaching, tissue necrosis and even plant death. It was widely applied in cotton, soybean, and tobacco. Sethoxydim disturbs fatty acid biosynthesis and disrupts the photosynthesis process in the plants of Poacea [6,7]. Under sethoxydim stress, the fresh and dry weight and chlorophyll contents of rape were reduced [8], and the lipid content and fatty acid synthesis activity of soybean were reduced [9]. In addition, the ACCase inhibitor decreased the expression of the ACCase gene in large crabgrass [10].

Foxtail millet has a high sensitivity to herbicides, making it difficult to find safer and more reliable herbicides. Foxtail millet is sensitive to many herbicides, such as nicosulfuron [11,12], sigma broad [13], prometryne and metolachlor [14]. The Zhangzagu series of hybrid foxtail millet is widely cultivated in large areas in Shanxi and Hebei, China, and it exhibits broad adaptability, excellent quality and high yield [15]. Additionally, the application of 22.5 g ai ha^–1^ of tribenuron-methyl at the post-emergence stage was found to be relatively safe for Zhangzagu 10 and did not affect its yield or grain quality [16]. Zhangzagu varieties exhibit a certain tolerance to sethoxydim, but high-concentration treatment still significantly inhibits their yield and other key agronomic traits. Therefore, systematic research on the regulatory mechanisms of sethoxydim on the photosynthetic and antioxidant systems of Zhangzagu is of significant scientific importance for elucidating the physiological response patterns and adaptive strategies of crops under herbicide stress.

C4 photosynthesis plants possess a broad adaptive capacity [17] and can mitigate oxidative stress-induced damage by regulating the synthesis and activity of photosynthetic enzymes [18]. For example, the activity of C4 photosynthetic enzymes in maize can rapidly recover after treatment with rimsulfuron and imazethapyr, revealing its active detoxification and metabolic repair mechanisms [19,20]. C4 photosynthetic enzymes, including NADP-malic enzyme (NADP-ME), phosphoenolpyruvate carboxylase (PEPC), pyruvate, phosphate dikinase (PPDK) and NADP-malic dehydrogenase (NADP-MDH), participate in the assimilation process of CO_2_ in C4 photosynthesis [21]. Although wheat is classified as a C3 plant, it also possesses C4 pathway enzymes, and under drought conditions, the activity of photosynthetic enzymes (PEPC, NADP-ME, NADP-MDH and PPDK) in spike organs significantly increases [22,23]. Under nicosulfuron treatment, the C4 photosynthetic enzyme activities and transcript levels in the nicosulfuron-tolerant maize line HK301 significantly increased, thereby enhancing its tolerance to nicosulfuron [24].

Once subjected to adverse stress, reactive oxygen species (ROS) in plants damage the lipids in biological membranes, oxidize proteins, inactivate enzymes and disrupt the normal metabolism of cells. Examples include hydrogen peroxide (H_2_O_2_) and superoxide anion (O_2_^·^−^^) [25,26]. Nevertheless, enzymes like superoxide dismutase (SOD), peroxidase (POD) and catalase (CAT) can eliminate the free radicals and reactive oxygen species generated during the metabolic process, thereby safeguarding cells from harm [27]. Ma et al. found that pyrazosulfuron-methyl inhibited the growth of foxtail millet while inducing an increase in antioxidant enzyme activities and a decrease in antioxidant substance contents [28]. Some studies have also shown that as the concentration of atrazine increases, the activities of ascorbate peroxidase and peroxidase are enhanced, while the activities of catalase and superoxide dismutase are decreased [25].

This study aims to systematically elucidate the effects of different sethoxydim concentrations on agronomic traits, photosynthetic parameters, key carbon assimilation enzyme activities, hydrogen peroxide (H_2_O_2_) and superoxide anion (O_2_^·^−^^) contents, soluble sugar and protein levels, as well as antioxidant enzyme activities in Zhangzagu 10. By detecting the expression levels of key genes related to carbon assimilation and antioxidant enzymes, it reveals the physiological and genetic-level changes in foxtail millet caused by sethoxydim through inhibition of fatty acid synthesis. The research findings will provide a theoretical basis for clarifying the herbicide resistance mechanisms in foxtail millet.

## 2. Materials and Methods

### 2.1. Plant Materials and Experimental Setup

Sethoxydim (12.5%, EC) was bought from Osaka Soda CO., Ltd., (Osaka, Japan). Foxtail millet seeds of Zhangzagu 10 were obtained from Hebei Universe Agriculture Technology Co., Ltd. (Shijiazhuang, China).

The pot experiment was conducted in a greenhouse under controlled conditions of 25 °C, a 16 h light/8 h dark photoperiod, a light intensity of 100 μmol m^−2^ s^−1^ and 40% relative humidity. The experiment adopted a completely randomized design with four replicates. The seeds were sown in plastic pots with a diameter of 20 cm filled with nutrient soil. Herbicide treatments were applied at the eight-leaf stage, with sethoxydim dose gradients set to 0 (control, CK), 0.75 (T1), 1.5 (T2, the recommended dosage) [29], 3.0 (T3) and 6.0 (T4) L ai ha^−1^, and the plants were treated using 3WP-2000 Bioassay Spray Tower (developed by Nanjing Institute of Agricultural Mechanization, Ministry of Agriculture and Rural Affairs, Nanjing, China). Measurements of plant height, leaf area, fresh weight and other parameters in foxtail millet were conducted 3, 6 and 12 days after sethoxydim treatment (DAT). Fresh leaf samples were collected at the same time points and stored at −80 °C for subsequent analysis.

### 2.2. Measurement of Plant Height, Leaf Area and Fresh Weight

The plant height was measured with a ruler. The leaf area was measured with a leaf area detecting device (YMJ-B, Zhejiang Top Cloud-agri Technology Co., Ltd., Hangzhou, China). The fresh weight of plants was weighed using an analytical balance.

### 2.3. Analysis of Photosynthetic Gas Exchange Parameters

The net photosynthetic rate (*Pn*), stomatal conductance (*Gs*) and transpiration rate (*Tr*) were detected via an open-flow gas exchange device (Li-6800, LI-COR, Lincoln, NE, USA) in the foxtail millet leaves during 9:30 to 11:00 am. The photon flux density (PFD) was 800 μmol m^−2^ s^−1^, the CO_2_ content was 400 μmol mol^−1^ and data was recorded when *Pn* was stable.

### 2.4. Determination of Chlorophyll Content

Chlorophyll a (Chl a), chlorophyll b (Chl b) and total chlorophyll (Chl a + b) were measured following Guo et al.’s method [30]. The fresh leaves of foxtail millet (0.1 g) were soaked in 10 mL of alcohol (96%, *v*/*v*) and stored without light for 24 h. The optical density (OD) value of supernatants was detected at 649 and 665 nm via a UV 2400 ultraviolet-visible spectrophotometer (Sunny Hengping Instrument, LLC, Shanghai, China).

### 2.5. Assay of Chlorophyll Fluorescence and P700 Parameters

Chlorophyll fluorescence and *P700* parameters were detected via Dual-PAM-100 (WALZ, Effeltrich, Germany). Prior to measurement, the treated seedlings were dark-adapted for 30 min. Subsequently, parameters related to photosystem II (PSII) were determined and calculated following the method described by Shimakawa [31], including minimal fluorescence (*Fo*), maximal fluorescence (*Fm*), PSII potential activity (*Fv/Fo*), maximum quantum yield of PSII (*Fv/Fm*) and photochemical quenching (*qP*). Meanwhile, parameters related to photosystem I (PSI) were also determined and calculated according to the same method, including the amount of functional PSI complex (*Pm*), PSI actual photochemical efficiency (*Y(I)*), PSI relative electron transport rate (*ETR(I)*) and the non-photochemical quantum yield due to PSI acceptor-side limitation (*Y(NA)*).

### 2.6. Measurement of Photosynthetic Enzyme Activities

Fresh leaves (0.2 g) were placed into pre-cooled extraction solution (100 mM Tris-HCl, 7 mM mercaptoethanol, 1 mM EDTA, 50% glycerin and 1% PVP [pH 8.2]) and ground until homogenized. The homogenate was centrifuged at 15,000× *g* for 20 min at 4 °C, and the supernatant was collected to determine the activity of ribulose-1,5-bisphosphate carboxylase/oxygenase (Rubisco), phosphoenolpyruvate carboxylase (PEPC), NADP-malic dehydrogenase (NADP-MDH), NADP-malic enzyme (NADP-ME) and pyruvate orthophosphate dikinase (PPDK).

Rubisco (EC 4.1.1.39) activity was spectrophotometrically determined via Lilley’s method [32] with some minor modifications; the reaction system included 1.4 mL 100 mM Tris-HCl (0.4 mM EDTA-Na_2_ [pH 7.8]), 0.2 mL 5 mM ATP, 0.2 mL 0.2 mM NaHCO_3_, 0.2 mL 5 mM NADH, 0.1 mL 160 U creatine phosphokinase, 0.1 mL 160 U glyceraldehyde-3 P-dehydrogenase, 0.2 mL 50 mM phosphocreatine, 0.1 mL 160 U Phosphoglycerate kinase PGK, 0.1 mL supernatant and 0.3 mL ddH_2_O. Finally, 0.1 mL 10 mM RuBP solution was added, and enzyme activity was determined spectrophotometrically, with the OD detected at 340 nm; data was collected every 30 s for 2 min.

PEPC (EC 4.1.1.31) activity was determined using Chen’s method [33]. The reaction system of PEPC included 1 mL 100 mM Tris-HCl (pH 8.2), 0.1 mL 5 mM NADH, 0.1 mL 10.5 U MDH, 0.1 mL 100 mM NaHCO_3_, 0.1 mL supernatant, 1.5 mL ddH_2_O and 0.1 mL 40 mM PEP. Start timing after adding PEP, record the OD value at 340 nm every 30 s and time for 2 min. The reference is the system without PEP.

NADP-ME (EC 1.1.1.40) activity was detected following the approach described by Sayre [34], with slight modifications. Reaction mixtures contained 1.0 mL 150 mM Tris-HCl (60 mM MnCl_2_ and 1.5 mM EDTA-Na_2_, pH 8.0), 0.1 mL 150 mM DTT, 0.1 mL 12 mM NADP^+^, 0.1 mL supernatant and 1.6 mL ddH_2_O. The reaction was initiated by adding 0.1 mL of 150 mM L^−1^ malate, and the OD value was detected at 340 nm; data was collected every 30 s for 2 min.

PPDK (EC 2.7.9.1) activity was determined using Ting’s method [35]; the reaction system of PPDK included 1.0 mL 150 mM Tris-HCl (18 mM MgSO_4_, pH 8.3), 0.1 mL 300 mM DTT, 0.1 mL 4.5 mM NADH, 0.1 mL 30 mM AMP, 0.1 mL 12 mM LPEP, 0.1 mL 60 U LDH, 0.1 mL supernatant, 1.3 mL ddH_2_O and 0.1 mL 30 mM sodium pyrophosphate anhydrous. Start timing after adding sodium pyrophosphate anhydrous and record the OD value at 340 nm every 30 s for 2 min.

### 2.7. Measurement of Soluble Sugar and Soluble Protein

Total soluble sugars were extracted from the youngest leaf and detected via the anthrone reagent method, as used by Yemm [36] with a UV–visible spectrophotometer. The quantity of it was expressed as µg soluble sugars g^−1^ fresh matter (fm). D Glucose was used as a standard.

During the experiment, the soluble protein content was detected via the Coomassie Brilliant Blue G-250 staining method [37]. The corresponding experimental process is as follows: collect fresh foxtail millet leaves (2.0 g), add 5 mL of distilled water, grind for 1 min, let it stand for half an hour and centrifuge at high speed for 15 min. Collect 2 mL of supernatant and add 8 mL of distilled water to it as a sample. Take 0.1 mL of the sample and add it to 5 mL of Coomassie Brilliant Blue G-250 dye. Mix thoroughly and incubate for 5 min. Perform absorbance detection at 595 nm and record relevant data.

### 2.8. Measurement of Antioxidant Enzyme Activities

According to the TBA method described by Heath and Packer, lipid peroxidation levels are measured by the amount of MDA [28]. Collect fresh foxtail millet leaves (0.5 g), cut them into small pieces, homogenize them with 5% trichloroacetic acid and then centrifuge at high speed for 10 min. Add 2 mL of extract to 2 mL of 0.6% TBA, mix thoroughly and treat in a boiling water bath for 10 min. After removing the sample, place it in a photometer and test at 532, 600 and 452 nm to determine the absorbance.

Fresh foxtail millet leaves (0.5 g) were homogenized in 5 mL PBS (0.1 mol/L and pH 7.8) and centrifuged at 10,000× *g* for 20 min at 4 °C, and the supernatant was applied to detect SOD and POD levels. The activity of SOD was detected through the nitro blue tetrazolium (NBT) method of Ries [38]. One unit of SOD was defined as the amount of enzyme required to cause 50% inhibition of the reduction in NBT as monitored at 560 nm. POD activity was detected based on Hammer Schmidt and others [39] by calculating the guaiacol oxidation rate at 470 nm. CAT activity was determined according to the instructions of the CC3221 kit (Beijing Bio-box Co., Ltd., Beijing, China).

### 2.9. Measurement of H_2_O_2_ Content and O_2_^·^−^^Content

The H_2_O_2_ content was determined according to the instructions for the AKAO009C kit (Beijing Bio-box Co., Ltd., Beijing, China). The superoxide anion (O_2_^·^−^^) production rate is determined using the method described by Wang and Luo [40], with some modifications; it is determined by monitoring the formation of nitrite from hydroxylamine in the presence of a superoxide anion (O_2_^·^−^^). Take fresh tissue (0.5 g), homogenize it with 1.5 mL of 50 mmol/L potassium phosphate buffer (pH 7.8) containing 1% (*w*/*v*) polyvinylpyrrolidone at 0 °C and then centrifuge at 5000× *g* and 4 °C for 15 min. Take 1 mL of the obtained supernatant, mix it with 0.9 mL of 50 mmol/L potassium phosphate buffer (pH 7.8) and 0.1 mL of 10 mmol/L hydroxylamine hydrochloride and then incubate at 25 °C for 30 min. Add 1 mL of the incubated solution to 1 mL of 17 mmol/L 3-aminobenzenesulfonic acid and 1 mL of 7 mmol/L 1-naphthylamine, and then let it stand at 25 °C for another 20 min. Record the absorbance at 530 nm. Use the standard curve of nitrite (NO_2_^−^) to calculate the content of superoxide anion (O_2_^·^−^^) according to the reaction equation of superoxide anion (O_2_^·^−^^) and hydroxylamine. The production rate of superoxide anion (O_2_^·^−^^) is expressed as μmol g^−1^ min^−1^ on a fresh weight basis.

### 2.10. Experimental Verification of Related Gene Expression Level by qRT-PCR

Total RNA was extracted from Zhangzagu leaves under the conditions described in Section 2.1 using the Trizol reagent kit AG21101 (Accurate Biology, Changsha, China). Subsequently, the RNA was reverse-transcribed into cDNA using the Evo M-MLV Reverse Transcription Kit AG11705 (Accurate Biology, Changsha, China). Real-time quantitative PCR (RT-qPCR) was performed using SYBR GreenPro Taq HS Premix AG11701 (Accurate Biology, Changsha, China). In the real-time quantitative PCR analysis, the primer for *SiActin* (*Seita.7G294000*) was referenced from Zhao et al. [41], while specific primers for all other target genes were designed and synthesized using Premier 5 software. The RT-qPCR reactions were conducted using a Bio-Rad CFX system and the relative expression levels were calculated using the 2^−∆∆Ct^ method. Three independent biological replicates were performed for the experiment. The specific experimental procedures are as follows: (1) Preparation of the reaction mixture: The amplification system had a total volume of 20 μL, containing 10 μL of 2× SYBR Green Pro Taq HS Premix, 0.4 μL of each of the forward and reverse primers, 2 μL of cDNA and 7.2 μL of RNase-free water. (2) RT-qPCR program: 95 °C for 30 s, followed by 40 cycles of 95 °C for 5 s and 59 °C for 30 s. The sequences of the specific primers used are listed in Table 1.

### 2.11. Statistical Analyses

Statistical analysis and graph plotting were performed using IBM SPSS Statistics 27 and OriginPro 2024 (OriginLab Corporation, Northampton, MA, USA). Data obtained from biological replicate experiments are presented as the mean ± standard error of the mean (Mean ± SEM). Significant differences between groups were determined via Tukey’s HSD test for multiple comparisons. Pearson correlation analysis was conducted using OriginPro 2024 software to assess the relationships between key physiological and biochemical parameters. A statistical significance threshold was set at *p* < 0.05. Different lowercase letters in the figures indicate statistically significant differences among various treatments at the same time point.

## 3. Results

### 3.1. Effect of Sethoxydim on Agronomic Traits of Foxtail Millet and Phenotypic Schematic Diagram

**Figure 1:**
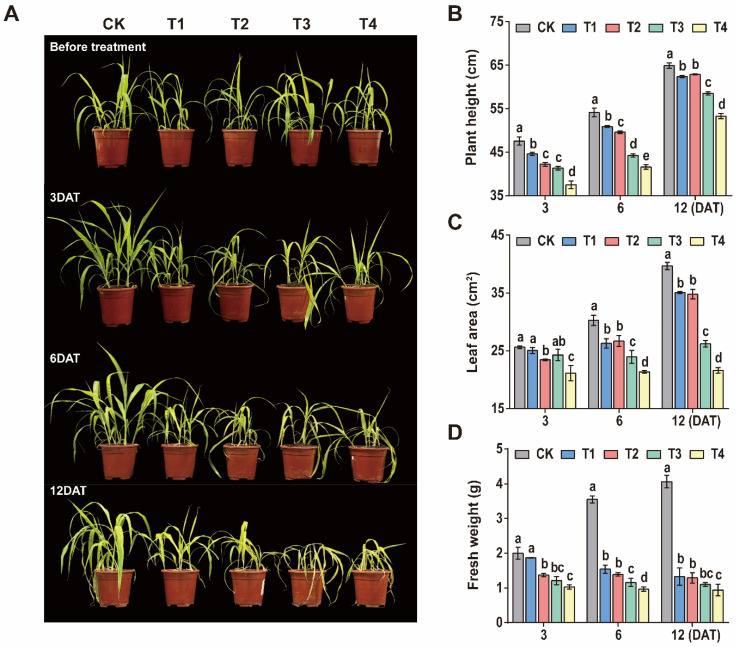
**Effect of sethoxydim on agronomic traits of foxtail millet.** (**A**) Schematic diagram of the growth status of Zhangzagu at different time points (3, 6, and 12 days) under different treatments (CK, T1, T2, T3, and T4); (**B**) plant height; (**C**) leaf area; (**D**) fresh weight. Note: Different letters in the figure indicate that the mean values of different treatments at the same time point are significantly different (Tukey’s HSD test, *p* ≤ 0.05).

With the increase in sethoxydim dosage, the plant height, leaf area and fresh weight of the plant reduced (Figure 1). Plant height significantly decreased with higher sethoxydim application rates, and this inhibitory effect persisted throughout the subsequent growth stages without obvious recovery. At 6 days after treatment (6 DAT), plant height in the T1, T2, T3 and T4 treatment groups decreased by 5.9%, 8.4%, 18.2% and 23.1%, respectively, compared to the control group (Figure 1B). At 3 DAT, compared to the control group (CK), the reductions in leaf area under the T1 and T3 treatments were not significant, while the T2 and T4 treatments resulted in significant decreases of 8.5% and 17.5%, respectively. By 6 DAT, the leaf area reductions in the treatment groups (T1, T2, T3 and T4) were 13.3%, 11.8%, 8.7% and 29.4%, respectively. At 12 DAT, the reductions in leaf area further increased to 11.7%, 12.3%, 25.2% and 45.5%, respectively (Figure 1C). At 6 DAT, the fresh weight of the T1, T2, T3 and T4 treatment groups decreased significantly compared to CK, with reductions of 56.6%, 60.8%, 67.3% and 72.7%. By 12 DAT, the fresh weight reductions continued to increase, reaching 67.2%, 68.2%, 72.9% and 76.8%, respectively (Figure 1D).

### 3.2. Effect of Sethoxydim on Photosynthetic Gas Exchange Parameters of Foxtail Millet

**Figure 2:**
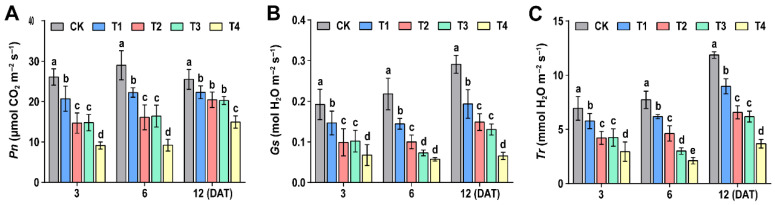
**Effects of sethoxydim on photosynthetic gas exchange parameters of foxtail millet**. (**A**) Net photosynthetic rate (*Pn*); (**B**) stomatal conductance (*Gs*); (**C**) transpiration rate (*Tr*). Note: Different letters in the figure indicate that the mean values of different treatments at the same time point are significantly different (Tukey’s HSD test, *p* ≤ 0.05).

Under the sethoxydim stress, the *Pn*, *Gs* and *Tr* of foxtail millet were significantly reduced, and there was no obvious recovery with the prolongation of time after applying sethoxydim (Figure 2). At 6 days after treatment (6 DAT), compared to the control group (CK), the decreases in net photosynthetic rate for the treatment groups (T1, T2, T3, and T4) were 23.3%, 44.4%, 43.4% and 68.2%, respectively. The stomatal conductance and transpiration rate in the T2 treatment group were also consistently and significantly inhibited, decreasing by 48.5% and 9.4% at 3 DAT; 54% and 40.1% at 6 DAT; and 48.8% and 44.6% at 12 DAT, respectively.

### 3.3. Effect of Sethoxydim on Photosynthetic Pigment Contents of Foxtail Millet

**Figure 3:**
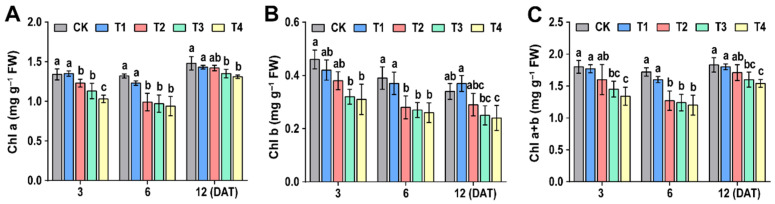
**Effect of photosynthetic pigment contents of foxtail millet**. (**A**) Chlorophyll a (Chl a); (**B**) chlorophyll b (Chl b); (**C**) total chlorophyll (Chl a + b). Note: Different letters in the figure indicate that the mean values of different treatments at the same time point are significantly different (Tukey’s HSD test, *p* ≤ 0.05).

As shown in Figure 3, the contents of chl a, chl b and chl a + b in foxtail millet leaves decreased with increasing sethoxydim dose. Among these, both chlorophyll a and total chlorophyll content were most significantly inhibited at 6 days after treatment (6 DAT). Chlorophyll a decreased by 6.8%, 25.0%, 26.5% and 28.8% under T1, T2, T3 and T4 treatments, respectively. The reduction in total chlorophyll followed a similar pattern, with decreases of 6.9%, 26.2%, 27.9% and 30.2%, respectively. Chlorophyll b content also decreased significantly at both 3 DAT and 6 DAT: at 3 DAT, it declined by 8.7%, 17.4%, 30.4% and 32.6% under T1 to T4 treatments, respectively; by 6 DAT, the reductions further increased to 5.1%, 28.2%, 30.8% and 33.3%. All indicators showed a dose-dependent decreasing trend.

### 3.4. Effect of Sethoxydim on Chlorophyll Fluorescence and P700 Parameters of Foxtail Millet

**Figure 4:**
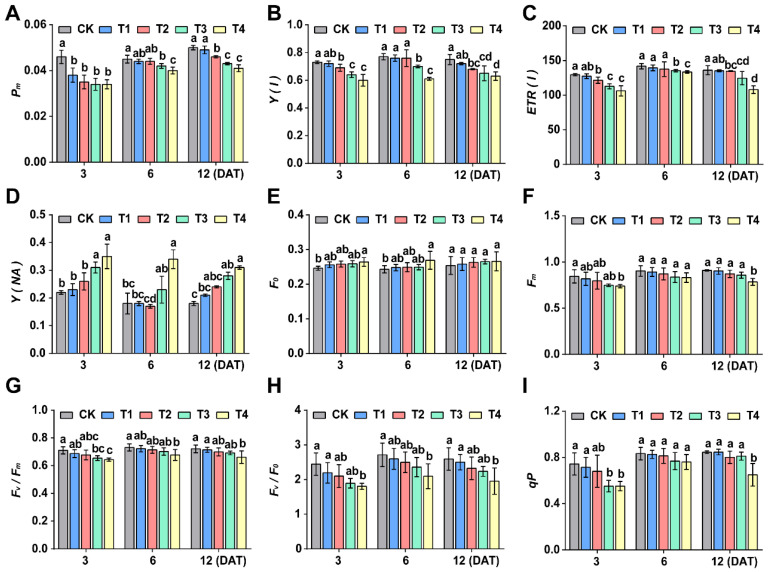
**Effect of chlorophyll fluorescence and P700 parameters of foxtail millet.** (**A**) PSI complex (*Pm*); (**B**) PSI actual photochemical efficiency (*YI*); (**C**) relative electron transfer rate of PSI (*ETR(I)*); (**D**) nonphotochemical quantum yield due to PSI acceptor side limitation (*NA*); (**E**) minimal fluorescence (*Fo*); (**F**) maximal fluorescence (*Fm*); (**G**) PSII potential activity (*Fv/Fo*); (**H**) maximum quantum yield of PSII (*Fv/Fm*); (**I**) photochemical quenching (*Qp*). Note: Different letters in the figure indicate that the mean values of different treatments at the same time point are significantly different (Tukey’s HSD test, *p* ≤ 0.05).

At different time points (3, 6 and 12 days), the parameters, including maximum photosynthetic capacity (*Pm*) (Figure 4A), effective quantum yield *Y(I)* (Figure 4B), relative electron transport rate *ETR(I)* (Figure 4C), maximum fluorescence *Fm* (Figure 4F), maximum photochemical efficiency *Fv/Fm* (Figure 4G), potential photochemical activity *Fv/F*_0_ (Figure 4H), and photochemical quenching coefficient *qP* (Figure 4I), all exhibited a declining trend with increasing sethoxydim dosage. Compared to the control group, foxtail millet seedlings treated with sethoxydim (T2, T3 and T4 treatment groups) showed significant differences in *Pm* (Figure 4A), *Y(I)* (Figure 4B) and *ETR(I)* (Figure 4C) at all time points, with the most pronounced differences observed on day 3 after treatment. At 3 days after treatment, the maximum photosynthetic capacity (*Pm*) (Figure 4A) in the T2, T3 and T4 treatment groups decreased by 23.9%, 26% and 26%, respectively. The effective quantum yield *Y(I)* decreased by 5.5%, 12.2% and 17.8%, while the relative electron transport rate *ETR(I)* (Figure 4C) decreased by 6.1%, 12.9% and 17.9%, respectively. In contrast, non-photochemical quenching *Y(NA)* (Figure 4D) exhibited an upward trend with increasing treatment dose: *Y(NA)* increased by 40.9% and 35.7% in the T3 group at 3 and 12 DAT, respectively, while in the T4 group, it rose by 59.1%, 88.9% and 72.2% at 3, 6 and 12 DAT, respectively. Meanwhile, initial fluorescence *F_0_* (Figure 4E) also showed an increasing trend with higher sethoxydim concentration.

### 3.5. Effect of Sethoxydim on Photosynthetic Enzyme Activities of Foxtail Millet

**Figure 5:**
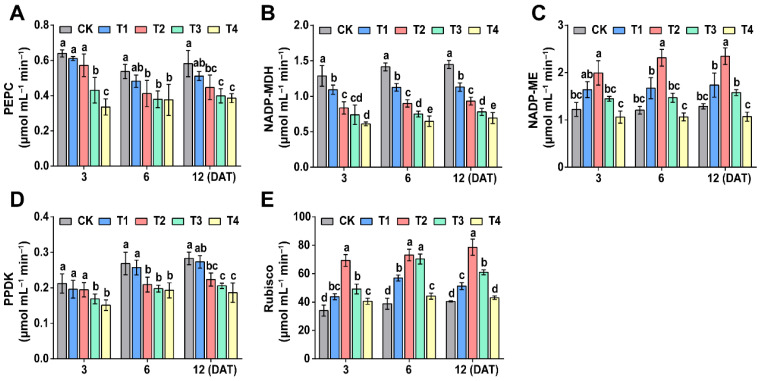
**Effect of photosynthetic enzymes activities of foxtail millet.** (**A**) Phosphoenolpyruvate carboxylase (PEPC); (**B**) NADP-malate dehydrogenase (NADP-MDH); (**C**) NADP-malic enzyme (NADP-ME); (**D**) pyruvate orthophosphate dikinase (PPDK); (**E**) Rubisco. Note: Different letters in the figure indicate that the mean values of different treatments at the same time point are significantly different (Tukey’s HSD test, *p* ≤ 0.05).

With increasing sethoxydim dose, the PEPC, NADP-MDH and PPDK activities decreased and the NADP-ME and Rubisco activities, which were initially enhanced, subsequently reduced with the prolongation of time after applying sethoxydim (Figure 5). Under T2 treatment, PEPC activity decreased by 10.6%, 23.4% and 23.2% compared to the control at 3, 6 and 12 days after treatment (DAT), respectively. The inhibitory effect on PEPC activity was more pronounced under T4 treatment at 3 DAT, with a reduction of 47.6% relative to the control (Figure 5A). NADP-MDH activity under T2 treatment declined by 35.0%, 20.4% and 35.6% at 3, 6 and 12 DAT, respectively. Greater reductions were observed under the T3 and T4 treatments, with decreases of 35.0%, 36.4% and 46.2% under T3, and 52.5%, 54.2% and 52.1% under T4 at the corresponding time points (Figure 5B). PPDK activity showed no significant difference from the control under T1 treatment; however, under T2 treatment, it decreased by 8.3%, 22.1% and 21.0% at 6 and 12 DAT, respectively. The most substantial decrease, 34.1%, occurred under T4 treatment at 12 DAT (Figure 5D). In contrast, NADP-ME activity increased by 63.2%, 92.0% and 82.5% under T2 treatment at 3, 6 and 12 DAT, respectively, but decreased under T3 and T4 treatments (Figure 5C). Similarly, Rubisco activity rose by 103.8%, 88.7% and 94.1% under T2 treatment at 3, 6 and 12 DAT, respectively, whereas it declined under T3 and T4 treatments (Figure 5E).

### 3.6. Effect of Sethoxydim on Soluble Sugar and Soluble Protein Contents of Foxtail Millet

**Figure 6:**
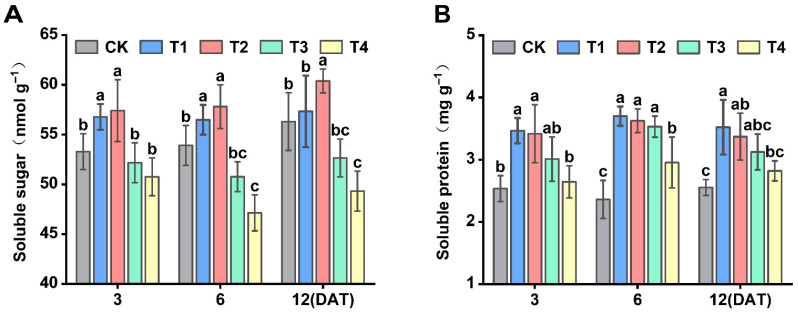
**Effect of soluble sugar and soluble protein contents of foxtail millet.** (**A**) Soluble sugar; (**B**) soluble protein. Note: Different letters in the figure indicate that the mean values of different treatments at the same time point are significantly different (Tukey’s HSD test, *p* ≤ 0.05).

With the increase in sethoxydim dosage, the total soluble sugar and protein contents of foxtail millet were first enhanced and then decreased (Figure 6). The soluble sugar content reached its highest level under the T2 treatment, increasing by 7.7%, 7.2% and 7.3% compared to the control (CK) at 3, 6 and 12 days after treatment (DAT), respectively. Under the T1 treatment, it also showed an increasing trend, with increases of 6.5%, 4.8% and 1.8%, respectively. In contrast, the T3 and T4 treatments exhibited inhibitory effects, decreasing by 2.1%, 5.8% and 6.5% for T3, and 4.7%, 12.6% and 12.4% for T4 compared to the CK (Figure 6A).

The soluble protein content was highest under the T1 treatment, showing significant increases of 36.7%, 56.6% and 37.9% compared to the CK at 3, 6 and 12 DAT, respectively. The T2 treatment also showed an upward trend, with increases of 34.8%, 53.5% and 32.0%, respectively. The increases under the T3 and T4 treatments were relatively lower, rising by 18.8%, 49.5% and 22.4% for T3, and 4.3%, 25.1% and 10.5% for T4 (Figure 6B).

### 3.7. Effect of Sethoxydim on Antioxidant Enzyme Activity of Foxtail Millet

**Figure 7:**
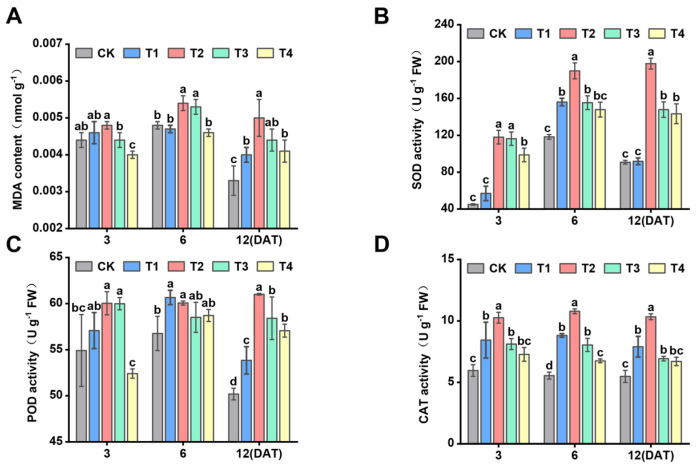
**Effect of antioxidant enzymes activity of foxtail millet**. (**A**) Malondialdehyde content (MDA); (**B**) superoxide dismutase activity (SOD); (**C**) peroxidase activity (POD); (**D**) catalase activity (CAT). Note: Different letters in the figure indicate that the mean values of different treatments at the same time point are significantly different (Tukey’s HSD test, *p* ≤ 0.05).

With increasing sethoxydim dose, the MDA, SOD, POD and CAT activities increased, then decreased with the prolongation of time after applying sethoxydim (Figure 7). The MDA activity under T2 treatment increased by 7.4%, 12.3% and 50.6% compared to the control at 3, 6 and 12 DAT, respectively (Figure 7A). SOD activity exhibited the highest increase on the third day after treatment under T2, T3 and T4 treatments, rising by 162.0%, 158.3% and 119.2%, respectively (Figure 7B). Compared to the control, the increase in POD activity on the 12th day after treatment was greater than that at the earlier two time points across T1, T2, T3 and T4 treatments. Specifically, POD activity increased by 7.3% (T1), 21.6% (T2), 16.4% (T3) and 13.7% (T4) relative to the control at 12 DAT, with these increments being notably higher than those observed at the previous time points (Figure 7C). CAT activity increased by 59.0%, 94.4%, 45.0% and 21.7% under T1, T2, T3 and T4 treatments, respectively, compared to the control at 6 DAT (Figure 7D).

### 3.8. Effect of Sethoxydim on H_2_O_2_ and O_2_^·^−^^ Content of Foxtail Millet

**Figure 8:**
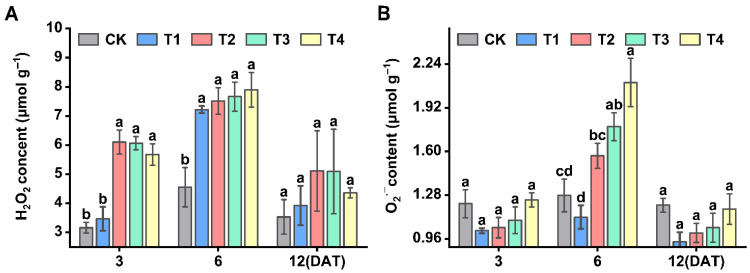
**Effect of reactive oxygen species (ROS) content of foxtail millet**. (**A**) H_2_O_2_ content; (**B**) O_2_^·^−^^content. Note: Different letters in the figure indicate that the mean values of different treatments at the same time point are significantly different (Tukey’s HSD test, *p* ≤ 0.05).

With the increase in the application amount of sethoxydim, the contents of hydrogen peroxide (H_2_O_2_) and superoxide anion (O_2_^·^−^^) gradually increased (Figure 8). Compared to the control group, the H_2_O_2_ content showed the greatest increase on 3 DAT under the T2 and T3 treatments, rising by 93.0% and 91.8%, respectively. On 6 DAT, the H_2_O_2_ content in all treatment groups (T1, T2, T3 and T4) increased by 58.4%, 64.9%, 68.3% and 73.5%, respectively (Figure 8A). Additionally, the O_2_^·^−^^ content was generally elevated on 12 DAT, with increases of 22.3%, 16.8%, 13.6% and 2.3% in the T1, T2, T3 and T4 treatment groups, respectively, compared to the control (Figure 8B).

### 3.9. Effect of Sethoxydim on Expression of Photosynthetic and Antioxidant Genes in Foxtail Millet

**Figure 9:**
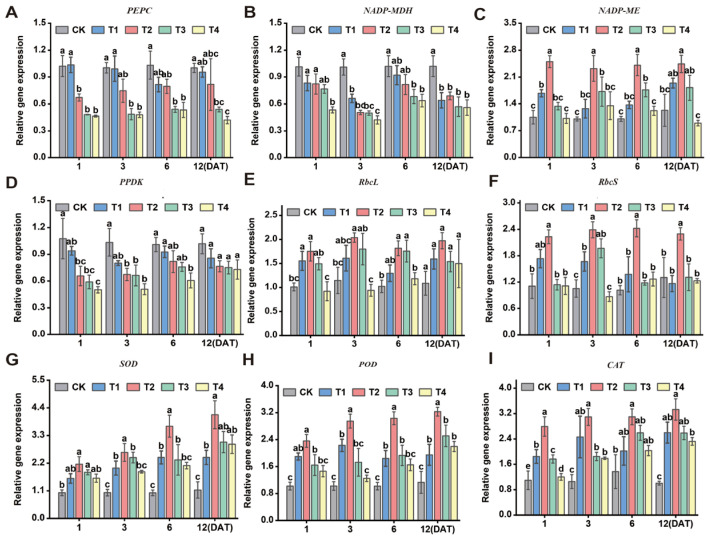
**Effect of sethoxydim on expression of photosynthetic and antioxidant genes in foxtail millet**. (**A**) PEPC; (**B**) NADP-MDH; (**C**) NADP-ME; (**D**) PPDK; (**E**) Rubisco large subunit—RbcL; (**F**) Rubisco small subunit—RbcS; (**G**) SOD; (**H**) POD; (**I**) CAT. Note: Different letters in the figure indicate that the mean values of different treatments at the same time point are significantly different (Tukey’s HSD test, *p* ≤ 0.05).

With the increase in the dose of sethoxydim, the expression levels of the genes encoding PEPC, NADP-MDH and PPDK decreased. However, the expression levels of the genes encoding NADP-ME and CAT first increased and reached the highest level under the T2 treatment. Subsequently, they decreased as the time after the application of sethoxydim was prolonged (Figure 9). On day 1 after treatment (DAT1), compared to the control, the T1 treatment increased the expression level of the gene encoding PEPC by 1.4%, while the T2, T3 and T4 treatments significantly reduced it by 34.4%, 52.9% and 54.6%, respectively. On the subsequent days 3, 6 and 12, the expression levels in all treatment groups continued to decline, and the degree of suppression increased with the dose (Figure 9A). The gene encoding NADP-MDH showed the most pronounced downward trend at 3 days after treatment (3 DAT), with decreases of 34.7%, 50.1%, 50.8% and 58.2% in the T1, T2, T3 and T4 treatment groups, respectively, compared to the control. Compared to the control, the expression of the gene encoding PPDK showed the most significant decrease at 1 and 3 days after treatment (DAT). On day 1 after sethoxydim treatment, the expression levels in the T1, T2, T3 and T4 treatment groups decreased by 22.0%, 19.1%, 24.6% and 47.5%, respectively. By day 3, the reductions further increased to 34.7%, 50.1%, 50.8% and 58.3% (Figure 9D). The expression level of the gene encoding NADP-ME showed the most significant increase at 6 days after sethoxydim treatment (6 DAT), with rises of 35.9%, 39.6%, 75.1% and 20.9% in the T1, T2, T3 and T4 treatment groups, respectively, compared to the control (Figure 9C). During the photosynthesis process of foxtail millet, the large subunit gene (*RbcL*) and the small subunit gene (*RbcS*) of Rubisco coordinately regulate the synthesis of this enzyme, thereby influencing the production of Rubisco. At 12 days after treatment (DAT12), the expression of the *RbcL* gene increased by 46.7%, 81.6%, 41.6% and 37.8% under the T1, T2, T3 and T4 treatments, respectively. In contrast, the expression of the *RbcS* gene showed the most significant increase at 3 DAT, rising by 58.3%, 126.9%, 86.8% and 17.6% under the corresponding treatments (Figure 9E,F). The gene expression levels of antioxidant enzymes (SOD, POD and CAT) were consistent with their enzymatic activity trends, peaking in the T2 group at 12 days after treatment (12 DAT). Specifically, the expression of the SOD encoding gene was upregulated by 114.6%, 264.8%, 168.6% and 161.2% under the T1, T2, T3 and T4 treatments, respectively; the POD encoding gene increased by 87.9%, 211.6%, 142.3% and 111.9%; and the CAT encoding gene rose by 158.3%, 230.9%, 157.3% and 130.9%.

### 3.10. Correlation Analysis

**Figure 10:**
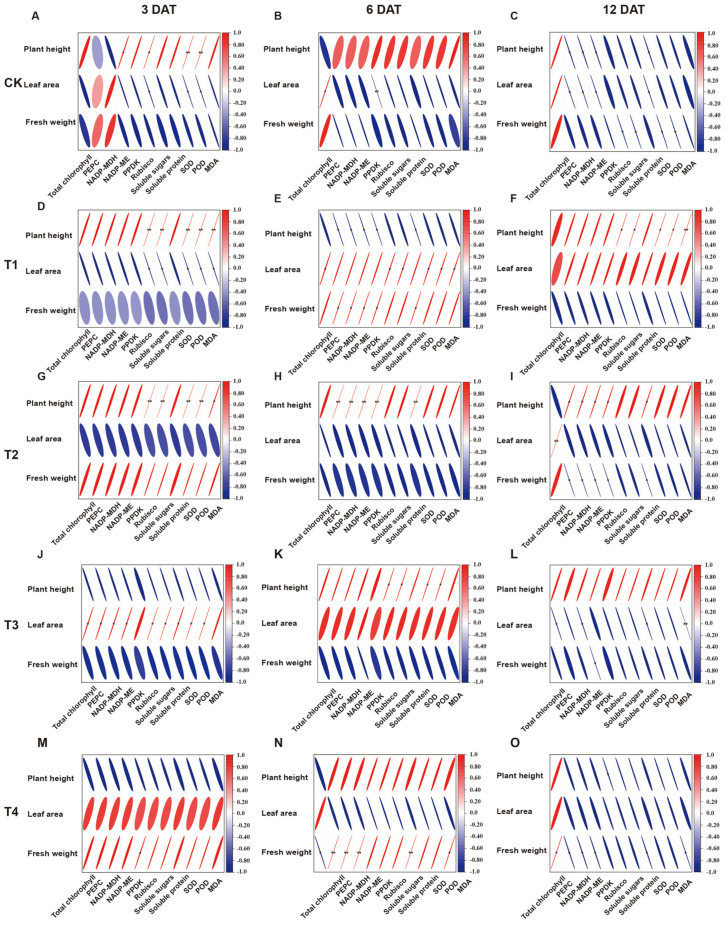
Relationships between plant height, leaf area, fresh weight, total chlorophyll content, PEPC, NADP-MDH, NADP-ME, PPDK, Rubisco, soluble protein, soluble sugar, SOD, POD and MDA. * and ** indicate that values are significant at *p* < 0.05 and *p* < 0.01, respectively. (**A**–**C**) Correlation analysis of the above indicators under CK treatment on the third, sixth and twelfth days; (**D**–**F**) correlation analysis of the above indicators under T1 treatment on the third, sixth and twelfth days; (**G**–**I**) correlation analysis of the above indicators under T2 treatment on the third, sixth and twelfth days; (**J**–**L**) correlation analysis of the above indicators under T3 treatment on the third, sixth and twelfth days; (**M**–**O**) correlation analysis of the above indicators under T4 treatment on the third, sixth and twelfth days.

Based on the results of correlation analysis (Figure 10), the associations among multiple dimensions of foxtail millet growth, photosynthetic physiology, carbon assimilation metabolism, and oxidative stress response under sethoxydim treatment at different time points and dosages were systematically evaluated. Plant height showed significant positive correlations (*p* < 0.01) with Rubisco enzyme activity, soluble sugar content, SOD activity, POD activity and MDA content on the third day after treatment under both the T1 and T2 treatments. Leaf area exhibited significant positive correlations (*p* < 0.05) with total chlorophyll content, PEPC, NADP-MDH, NADP-ME, Rubisco enzyme activity, soluble sugar content, soluble protein content, SOD activity and POD activity on the third and sixth days after treatment under T3 treatment, whereas under T2 treatment at the same time points, it showed negative correlations with the above indicators. Fresh weight showed significant negative correlations (*p* < 0.05) with PEPC, Rubisco enzyme activity, NADP-MDH, NADP-ME, PPDK, soluble sugar content, soluble protein content, SOD activity, POD activity and MDA content on the 12th day after treatment across all treatments (CK, T1, T2, T3, T4).

## 4. Discussions

Herbicides can cause certain damage to crops while controlling weeds in the field, affecting their normal growth and development. The agronomic traits of crops can visually reflect the harm caused by herbicides [42]. Previous studies have shown that quinclorac and bispyribac-sodium reduce rice plant height, while carfentrazone-ethyl not only inhibits plant height but also induces distinct phytotoxicity symptoms [43]. Metolachlor inhibits plant height and stem diameter in common buckwheat, which in turn leads to a reduction in yield [44]. This experiment found that spraying sethoxydim on Zhangzagu 10 at the eight-leaf stage significantly inhibited plant height, leaf area and fresh weight, with the inhibition intensifying as the application rate increased.

Photosynthetic parameters can effectively reflect the photosynthetic capacity of plants [45]. Parameters such as leaf net photosynthetic rate (*Pn*), stomatal conductance (*Gs*) and transpiration rate (*Tr*) work synergistically during photosynthesis to ensure the smooth progression of the process [46]. Following bentazon application, both the net assimilation rate and stomatal conductance in soybean decreased with increasing herbicide rates [47]. In this experiment, with increasing concentration of sethoxydim treatment, the net photosynthetic rate (*Pn*), stomatal conductance (*Gs*) and transpiration rate (*Tr*) of foxtail millet leaves decreased significantly, indicating that sethoxydim inhibited the photosynthetic process.

Photosynthetic pigments play a crucial role in the absorption and initial conversion of light energy, and their conversion efficiency directly affects the photosynthetic capacity and growth rate of plants [48]. Photosynthetic pigments can absorb light energy and promote the generation of nutrients and have a certain protective effect on chloroplasts [49]. Therefore, when analyzing the impact of stress factors on plant photosynthesis, the content of pigments can be used to determine. Soares et al. [50] found that after being affected by glyphosate, the total chlorophyll and photosynthetic pigment content in cucumber leaves significantly decreased. After applying 3-cyanobenzoic acid to maize, its photosynthetic parameters showed a declining trend, and growth was significantly inhibited [51]. The results of this experiment indicate that after spraying sethoxydim at the eight-leaf stage of foxtail millet, the contents of chlorophyll a, chlorophyll b and total chlorophyll all decreased to varying degrees. Furthermore, the trend of reduction in relative chlorophyll content corresponded with the decline in leaf net photosynthetic rate, suggesting that the decrease in relative chlorophyll content is one of the factors leading to the reduction in the net photosynthetic rate of foxtail millet.

During the light-dependent reactions, plants rely on the coordinated function of photosystem II (PSII) and photosystem I (PSI) to convert light energy into chemical energy and reducing power. However, this process is highly susceptible to abiotic stresses. When exposed to herbicides, although PSII continues to absorb light energy, this energy triggers the formation of chlorophyll triplet states, subsequently inducing the generation of reactive oxygen species (ROS), ultimately leading to damage in plant proteins and membrane structures. The photosynthetic efficiency, electron transport rate (ETR), actual quantum yield of PSII (ΦII), *Fv/Fm* ratio and photochemical quenching coefficient (*qP*) in rice were all significantly inhibited after treatment with bentazon [52]. According to the experiment result, with the increase in sethoxydim dose, *Pm*, *Y(I)* and *ETR(I)* continuously reduced, indicating that sethoxydim had an inhibitory effect on photosystem I and slowed down the electron transport. As the dose of sethoxydim increased, *Y(ND)* first increased and then decreased, suggesting that under certain sethoxydim stress, Zhangzagu 10 could achieve self-protection by increasing heat dissipation. The gradual increase in *Y(NA)* reflects that sethoxydim blocked the activity of key enzymes in that process, hindering photosynthetic carbon assimilation and leading to electron accumulation on the acceptor side of photosystem I. Chlorophyll fluorescence is a probe for photosynthesis, and chlorophyll fluorescence parameters can reflect the site of photosynthetic inhibition [53]. With the increase in sethoxydim dose, the *Fv/Fm*, *Fv/Fo*, *Y(II)* and *ETR(I)* of Zhangzagu 10 tended to decrease, indicating that sethoxydim inhibited the potential activity of the photosystem II reaction center, reduced light utilization capacity and decreased electron transport efficiency. With the increase in the applied dose, *qP* gradually decreased and *NPQ* gradually increased, indicating that sethoxydim inhibited the photosynthetic activity of foxtail millet, and the plant consumed more energy by accelerating heat dissipation to achieve self-protection. Reduced photosynthetic efficiency leads to inhibited biomass accumulation and growth rate, ultimately affecting crop yield.

The photosynthetic pathway of foxtail millet is the C_4_ NADP-ME type, with PEPC, NADP-MDH, NADP-ME, PPDK and Rubisco as the key enzymes in this process [3]. PEPC can fix low concentrations of CO_2_ in the environment and CO_2_ produced by respiration, enhancing photosynthesis; MDH converts oxaloacetate to malate; malate undergoes decarboxylation and reduction under the action of NADP-ME to generate CO_2_ and pyruvate. The generated CO_2_ re-enters the bundle sheath cells for the Calvin cycle, while pyruvate is transported back to the mesophyll cells. In the C_4_ photosynthetic pathway, the specific enzyme PPDK regenerates PEP, and PPDK is an important rate-limiting enzyme [54]. The activity of key enzymes in photosynthetic carbon assimilation is one of the non-stomatal limitations that affect plant photosynthesis. Studies have demonstrated that reduced photosynthetic activity leads to decreased crop yield [55,56]. The introduction of maize *pepc* and *ppdk* genes into wheat, either separately or in combination, enhanced the photosynthetic capacity of wheat, thereby increasing its biomass and grain yield [57,58]. Under glyphosate treatment, the upregulation of *rbcL* gene expression in sugarcane enhances carbon fixation and photosynthetic activity, thereby compensating for the herbicide-induced inhibition of the shikima [59]. According to the aforementioned results, both the activities of PEPC, NADP-MDH and PPDK showed a decreasing trend with increasing application rates of sethoxydim, which may be because sethoxydim inhibits the activity of acetyl-CoA carboxylase in the chloroplast [60], impeding fatty acid synthesis, thereby preventing the cellular membrane system (particularly the chloroplast membrane) from being renewed and repaired, which impairs the photosynthetic electron transport chain and leads to a sharp decline in ATP synthesis efficiency. Relevant studies have shown that appropriate stress can activate the malic enzyme gene, increasing its expression and enzyme activity [61]. The results of this study show that when the spraying dosage is low, the activity of NADP-ME increases; however, as the dosage increases, its activity instead declines, showing no significant difference compared to the control group. The trend in Rubisco activity is similar to that of NADP-ME: under low-concentration sethoxydim stress, Rubisco activity increases correspondingly to cope with the decline in phosphoenolpyruvate carboxylase activity and maintain normal photosynthesis, but its activity decreases at higher dosages [62].

In plant cells, soluble proteins are enzymes not specifically bound to the membrane system and can reflect various aspects of intracellular protein synthesis, denaturation and degradation [27]. Carbohydrate metabolism is a fundamental aspect of plant primary metabolism. Under stress conditions, the accumulation of sugars plays a crucial role in regulating osmotic balance and maintaining cellular enzymatic activity [63]. On one hand, stress can induce the synthesis of soluble proteins in leaves to maintain a lower osmotic potential [64]; on the other hand, a decline in the transport capacity of photosynthetic assimilates may lead to an increase in soluble sugar content [65]. The experimental results indicate that as the concentration of sethoxydim increases, the contents of both soluble proteins and soluble sugars in the leaves initially rise and then decline. Under low-dose treatment conditions, the increase in soluble sugar and soluble protein levels in foxtail millet leaves may result from compensatory growth responses triggered by the plant’s own regulatory mechanisms. In contrast, under high-dose stress, multiple factors such as damage to the chloroplast structure, reduced photosynthetic synthesis and increased respiratory consumption may collectively disrupt normal metabolism, ultimately leading to a decrease in the content of soluble sugars and soluble proteins.

As a product of lipid peroxidation, the level of MDA is associated with the oxidative stress level in plants under stress conditions. When plant tissues are subjected to herbicide stress, MDA reacts with proteins, inhibiting protein synthesis and destroying cell membrane, causing the leakage of intracellular substances [66]. Under Prima Forte 195 treatment, wheat plants experience oxidative stress, resulting in increased levels of MDA [67]. Following the application of carfentrazone-ethyl, the levels of MDA and H_2_O_2_ increased in the leaves of soybean, maize and cotton [67]. In this experiment, as the concentration of sethoxydim increased, the H_2_O_2_ content rose, while MDA levels initially increased and then decreased, and O_2_^·^−^^ showed the opposite trend. This indicates that the antioxidant system of Zhangzagu 10 seedlings can protect the plants from oxidative damage under low concentrations of sethoxydim treatment.

Plants have developed antioxidant enzyme systems and non-enzyme systems during long-term evolution to mitigate the harmful effects of ROS and maintain the integrity of membrane structures [68]. Superoxide dismutase (SOD), peroxidase (POD), catalase (CAT), ascorbate peroxidase (APX) and glutathione reductase (GR) all belong to antioxidant enzymes, and they work together to defend against cellular damage induced by reactive oxygen species [25]. Under bromoxynil treatment, wheat plants exhibited upregulation in the activities of SOD, CAT, GPX and GST enzymes, along with increased expression levels of glutathione synthase gene (*TaGS*) and glutathione peroxidase gene (*TaGPX*), while plant height, fresh weight and dry weight decreased [69]. In this study, as the concentration of sethoxydim increased, the activities of antioxidant enzymes in Zhangzagu 10 gradually increased, which could effectively scavenge excessive reactive oxygen species and the products of membrane lipid peroxidation. This maintained the membrane structures and the balance of reactive oxygen species in foxtail millet. As can be seen from the decrease in the contents of hydrogen peroxide (H_2_O_2_) and superoxide anion (O_2_^·−^), these enzymes alleviated the oxidative damage caused by sethoxydim. However, when the concentration of sethoxydim exceeded 3.0 L ai ha^−1^, the activities of antioxidant enzymes decreased, and they were unable to scavenge the free radicals in foxtail millet. The sharp increase in the contents of H_2_O_2_ and O_2_^·−^ also demonstrated this point.

Furthermore, under abiotic (herbicide) stress, cells initiate their defense response through the regulation of differential gene expression [70]. The coordinated regulation of photosynthetic efficiency and related enzymes determines the formation of crop yield and quality [71]. Ammonium stress significantly enhances the enzymatic activity of PEPC in sorghum roots, a phenomenon closely associated with the upregulation of the root-specific gene *PPC3*. Meanwhile, the expression of the *PPCK1* gene in leaves is induced to increase, thereby activating PEPC kinase and promoting the phosphorylation modification of photosynthetic PEPC under both light and dark conditions [72]. After treatment with nicosulfuron, the tolerant variety ‘HK301’ alleviates the phytotoxic effects induced by the herbicide by upregulating the expression of genes encoding SOD and CAT [73]. The results of this study indicate that with increasing concentrations of sethoxydim treatment, the expression levels of genes encoding PEPC, NADP-MDH and PPDK gradually decrease. Under low-concentration treatment conditions, the expression of genes encoding NADP-ME, Rubisco, SOD, POD and CAT is significantly upregulated, whereas high-concentration treatment significantly suppresses the expression of these genes. The changes in enzyme activity and the corresponding gene expression patterns show coordinated trends after sethoxydim treatment, but the underlying causal relationship still requires further elucidation through genetic or biochemical experiments. Correlation analysis indicates significant associations between photosynthetic parameters, antioxidant enzyme activity and plant agronomic traits; however, this relationship is not a simple linear cause-and-effect. With the increase in sethoxydim treatment dosage, the decline in agronomic traits may be the integrated manifestation of the dynamic interaction across temporal and spatial dimensions among multiple physiological processes, including reduced photosynthetic efficiency, increased metabolic consumption and intensified reactive oxygen species accumulation.

This study only measured the photosynthetic parameters and antioxidant indices of Zhangzagu foxtail millet under different concentrations of sethoxydim treatment. It did not extend to other foxtail millet varieties and lacked validation in field environments; thus, the generalizability and practical applicability of the conclusions are subject to certain limitations.

## 5. Conclusions

**Figure 11:**
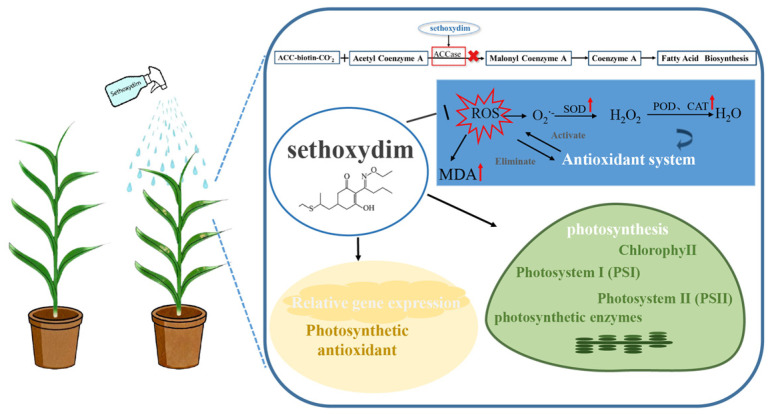
Schematic diagram of the mechanism of action of sethoxydim on foxtail millet.

Sethoxydim, as a highly selective systemic foliar herbicide, functions by inhibiting ACCase enzyme activity, thereby obstructing fatty acid synthesis and consequently preventing the formation of phospholipids and essential secondary metabolites in plants. This study investigates the effects of different doses of sethoxydim on the growth and development of ‘Zhangzagu’ foxtail millet, evaluating its safety based on agronomic traits, photosynthetic mechanisms and the antioxidant system. The findings provide a scientific basis for the safe, effective and rational application of sethoxydim in foxtail millet fields. The experimental results indicate that when the application rate of sethoxydim does not exceed 1.5 L ai ha^−1^, foxtail millet plants can alleviate herbicide-induced stress by enhancing photosynthetic efficiency, activating the antioxidant enzyme system and related gene expression and strengthening the scavenging capacity of reactive oxygen species (ROS). However, excessive application disrupts intracellular ROS balance, inducing oxidative stress. Accumulated ROS damages proteins and lipids, alters redox homeostasis and subsequently triggers photoinhibition, ultimately impairing the photosynthetic apparatus. This is manifested as decreased photosynthetic pigment content, reduced photosynthetic efficiency and downregulated activity and gene expression of key enzymes in both PSII and the C4 photosynthetic pathway. As a result, the growth and development of foxtail millet are inhibited, specifically reflected in reduced plant height, decreased leaf area and lower fresh weight (Figure 11).

## Figures and Tables

**Table 1:** List of primer sequences for photosynthetic and antioxidant enzyme genes in foxtail millet.

	Enzyme Name	Primer (5′-3′)
*Seita.7G294000*	β-Actin-F	CAGTGGACGCACAACAGGTAT
	β-Actin-R	AGCAAGGTCAAGACGGAG AAT
*Seita.4G175200*	PEPC-F PEPC-R	TGCGTGCTGGAATGAGTTACT CCAATAAGCATACATCCCGTG
*Seita.2G137100.1*	NADP-MDH-F NADP-MDH-R	ACGGCAAGCCAGCGAAAC AGTCGCCATCGCCCTTTG
*Seita.2G322000.1*	NADP-ME-F NADP-ME-R	GAGAGCTCTGTTTGCTATTTCG TCAAGGCTGCTTGCTTTATAAC
*Seita.3G247900.1*	PPDK-F	TCTCGAACGGCACGATGACC
	PPDK-R	CGGACTCGGAGCGAACCAC
*Seita.J020800.1*	Rubisco(rbcL)-F	TTGAAGAGGGTTCTGTTAC
	Rubisco(rbcL)-R	ACTTGGATACCGTGAGGC
*Seita.3G312200.1*	Rubisco(RBCS)-F	CACCTCCGTCGCTCCATT
	Rubisco(RBCS)-R	CGAACCCAACCTTGCTGAAC
*Seita.4G031200.1*	SOD-F	AGGGTGGTCTGTCTCGTA
	SOD-R	CCTGTGCTGCATTGTTAT
*Seita.1G022400.1*	POD-F	ACCTTGTTCACCGTCCCACC
	POD-R	CGTCCGTGTTGATGTCCAGAA
*Seita.1G117500.1*	CAT-F	ACTCCGACGACAAGATGCTGC
	CAT-R	GCGAACCTCCTCACGAACC

## Data Availability

The original contributions presented in this study are included in the article. Further inquiries can be directed to the corresponding authors.
